# Phosphorus in Cutaneous Diseases

**Published:** 1869-04

**Authors:** 


PHOSPHORUS IN CUTANEOUS DISEASE.—From *Journal of Cutaneous Medicine*, October.

Phosphorus, as a renovator of nerve-tissue and nerve force, has considerable claim to be considered a possibly useful remedy for a class of diseases which are due to debility and consequently to exhaustion of nerve power. To this class belong all the nutritive affections of the skin, and many of those of congestion.

Since our attention was first drawn to the virtues of phosphorus, we have employed it in lupus, in general alopecia, and in several cases of nerve irritability and nerve exhaustion, and with the most encouraging results. We have adopted as our formulæ one grain to four ounces of cod-liver oil, a teaspoonful a dose, directly after meals, three times a day; and one grain dissolved in a drachm and a half of suet, and divided into thirty pills; one three times a day at similar periods; and we have increased the strength of each formula, on repetition, to one grain and a half of phosphorus. We have met with no deterring symptoms, and where the remedy did not seem to be doing good, it was, in the opposite sense, negative. A delicate young lady afflicted with scrofulous lupus, mentioned the production of a feeling of warmth in her abdomen, and also in the feet and hands, which passed away in a few days; but this is the only immediate effect we have heretofore observed.

But the best results of the remedy were shown in the case of a young officer, sadly exhausted by residence in India and syphilitic neuralgia, the neuralgic attacks being accompanied with intense night sweats. The neuralgia and night sweats always yielded to the iodide of potassium, but the potash weakened him, and he was left prostrate for two or three weeks afterwards. On a late occasion he complained of a recurrence of his night sweats, with great debility and weariness; he was unable in the evening to take his place at the dinner table, and was obliged instead to go to bed. Although hot and perspiring in bed, his skin could not bear the chill of the open window; and his own account of himself was that his head ached, his back felt as if it were broken, and his legs refused to carry him. Under these circumstances, and seeing that he had no decided neuralgia, although complaining of three spots on the head, which were a little painful, but very tender to the touch, we hesitated to repeat the iodide of potassium, and prescribed the phosphorus pills, one to be taken three times a day, with a mixture of cinchona and sulphuric acid twice daily. The new remedy produced no apparent effect for a week, but after that time it acted like a charm. He felt generally strengthened; his previous sense of weariness disappeared, the night sweats ceased, he lost his susceptibility to taking cold, the tenderness of the scalp subsided, and he experienced an universal feeling of invigoration. He discovered also that the medicine, without knowing what it was, was in its action aphrodisiac. Comparing its influence on his health with the iodide of potassium, he remarked that he felt stronger after the use of the phosphorus; while the iodide, although it removed the immediate symptoms equally effectually, left him prostrate and weak.

